# The association between maternal dietary micronutrient intake and neonatal anthropometry – secondary analysis from the ROLO study

**DOI:** 10.1186/s12937-015-0095-z

**Published:** 2015-10-07

**Authors:** Mary K Horan, Ciara A McGowan, Eileen R Gibney, Jean M Donnelly, Fionnuala M McAuliffe

**Affiliations:** 1 UCD Obstetrics and Gynaecology, School of Medicine and Medical Science, University College Dublin, National Maternity Hospital, Dublin 2, Ireland; 2 UCD Institute of Food and Health, University College Dublin, Dublin 4, Ireland

**Keywords:** Maternal nutrition, Micronutrients, Pregnancy, Neonatal adiposity, Fetal programming

## Abstract

**Background:**

Micronutrients are necessary for fetal growth. However increasingly pregnant women are nutritionally replete and little is known about the effect of maternal micronutrient intakes on fetal adiposity in mothers with increased BMI. The aim of this study was to examine the association of maternal dietary micronutrient intake with neonatal size and adiposity in a cohort at risk of macrosomia.

**Methods:**

This was a cohort analysis of 554 infants from the ROLO study. Three day food diaries from each trimester were collected. Neonatal weight, length, circumferences and skinfold thicknesses were measured at birth. Multiple linear regression was used to identify associations between micronutrient intakes and neonatal anthropometry.

**Results:**

Birthweight was negatively associated with maternal trimester 3 vitamin D intake and positively associated with trimester 3 vitamin B12 intake R2adj 19.8 % (F = 13.19, *p* <0.001). Birth length was positively associated with trimester 3 magnesium intake R2adj 12.9 % (F = 8.06, *p* <0.001). In terms of neonatal central adiposity; abdominal circumference was positively associated with maternal trimester 3 retinol intake and negatively associated with trimester 3 vitamin E and selenium intake R2adj 11.9 % (F = 2.93, *p* = 0.002), waist:length ratio was negatively associated with trimester 3 magnesium intake R2adj 20.1 % (F = 3.92, *p* <0.001) and subscapular:triceps skinfold ratio was negatively associated with trimester 1 selenium intake R2adj7.2 % (F = 2.00, *p* = 0.047).

**Conclusions:**

Maternal micronutrient intake was associated with neonatal anthropometry even in women not at risk of malnutrition. Further research is necessary to determine optimal micronutrient intake in overweight and obese pregnant women.

**Trial registration:**

Current Controlled Trials ISRCTN54392969.

**Electronic supplementary material:**

The online version of this article (doi:10.1186/s12937-015-0095-z) contains supplementary material, which is available to authorized users.

## Introduction

Micronutrients are necessary for normal growth and development of the fetus and deficiencies have been found to be associated with intrauterine growth retardation and small for gestational age (SGA) infants [1, 2]. In developing countries, supplementation with single [3] or multiple [4] micronutrients has been found to increase birthweight and reduce risk of SGA. However in the developed world, where pregnant women are nutritionally replete and increasingly over-nourished with the risk of macrosomia (birthweight >4 kg or >95th centile) increasing, little is known about micronutrient intakes in pregnant women in relation to fetal size and adiposity. The prevalence of overweight and obesity has increased in women of childbearing age and is associated with increased levels of offspring macrosomia, childhood and later-life overweight and obesity which is thought to occur through metabolic programming [5]. In addition, levels of inflammation naturally increase during pregnancy, mediated by the placenta. The combination of pregnancy and maternal overweight or obesity leads to a chronic inflammatory environment which is thought to affect the fetus indirectly (as cytokines are negligibly directly transferred across the placenta) by increasing glucose and lipid availability [5, 6]. The increased availability of glucose and lipids results in increased fetal growth and fat deposition [6]. Micronutrients have many functions, for example some may be involved in drug-nutrient interactions, antioxidant processes, interactions with intercellular signalling proteins, transcriptional regulation, cell proliferation, platelet aggregation and monocyte adhesion as well as other as yet unknown functions [7, 8]. In addition, there appears to be a synergistic action of micronutrients in food both with each other and with other food components [9]. Finally, micronutrients may also act simply as markers of healthy overall diet and lifestyle confounding the relationship between their mechanisms of action and health outcomes [10]. Due to the multiple possible mechanisms of action, some of which have not fully been uncovered, and to the fact that micronutrients alone may not be resulting in certain health benefits with which they are associated, but rather the health behaviours their consumers undertake, the effect of dietary consumption of micronutrients is not yet fully understood and supplementation with these micronutrients does not always give the expected result [10].

Animal models have shown that micronutrient deficiency may increase offspring adiposity at birth. Antioxidant supplementation has been found by one study to reduce the effect of maternal obesity on neonatal adiposity in rats [11]. Another study found that maternal antioxidant supplementation also reduced the inflammatory destruction of murine fetal pancreatic β-cells reducing development of type II diabetes [12]. Vitamin D deficiency has similarly been found to be associated with increased risk of gestational diabetes in human studies [13] however human clinical trials are ongoing to determine causality. There remains a paucity of data on the effect of micronutrient intakes on human offspring adiposity, except in the prevention of low birthweight [14]. Therefore, the aim of this study was to examine the influence of maternal dietary micronutrient intake in pregnancy on neonatal weight, length and adiposity using a cohort at risk of macrosomia from the ROLO (Randomised cOntrol trial of LOw glycaemic index diet versus no dietary intervention to prevent recurrence of fetal macrosomia) study [15].

## Methods

Five hundred forty-two mother and infant pairs from the ROLO study were included in this analysis. The ROLO study was a randomised control trial of 800 secundigravid women with a previous macrosomic baby (>4 kg) randomised to receive low glycaemic index (GI) dietary advice versus usual care (no dietary advice) to reduce recurrence of macrosomia [16]. The ROLO study was carried out at the National Maternity Hospital, Ireland between January 2007 and January 2011 and detailed methodology and results have previously been published [15]. In brief; the primary outcome, a reduction in birthweight was not achieved and the secondary outcomes, a reduction in gestational weight gain and glucose intolerance, were achieved. Low GI dietary advice was given at week 14 of pregnancy while demographic, well-being and lifestyle questionnaires were returned by 28 weeks gestation. Three-day food diaries were completed at each trimester of pregnancy and used to determine the glycaemic index and glycaemic load of the mothers’ diets. The control group received routine antenatal care which did not involve dietary advice. This study was conducted according to the guidelines laid down in the Declaration of Helsinki and all procedures involving patients were approved by the National Maternity Hospital, Ireland ethics committee. Written informed maternal consent was obtained from all participants. The work presented here will use the dietary and anthropometric data from the ROLO study in order to carry out a secondary analysis examining the association of maternal micronutrient intake and neonatal size and adiposity.

### Inclusion and exclusion criteria

Participants were secundigravid women who had previously given birth to a macrosomic baby (>4 kg). They were required to have sufficient literacy and fluency in the English language to understand the intervention and be capable of completing questionnaires. Women were included if they were over 18 years old and free from underlying health conditions and if they had healthy, singleton pregnancies without any intrauterine growth abnormalities.

### Maternal demographics and lifestyle

Of the 800 participants of the ROLO study, 759 completed the original trial and had their infants’ anthropometry measured at birth. Of these, 542 completed and returned all questionnaires and food diaries. Questionnaires completed in the first half of pregnancy explored various background socioeconomic and socio-demographic, and lifestyle variables. Questions from SLAN (Survey of Lifestyle, Attitudes and Nutrition in Ireland) [17] relating to lifestyle habits were completed at this time, including questions on number of 20 min intervals of mild, moderate and strenuous physical activity per week and on number of days per week walking for over 30 min, on smoking and alcohol consumption, on educational attainment and on supplement use.

### Maternal and neonatal anthropometry

Maternal weight (kg), height (cm) and mid-upper arm circumference (cm) were measured at the first antenatal consultation and BMI was calculated. Maternal weight was also measured at each subsequent consultation and gestational weight gain was calculated.

Neonatal weight (kg), length (cm), mid-upper arm, abdominal, hip and thigh circumference (cm), and biceps, triceps, subscapular and thigh skinfold (mm) measurements were taken at birth. Weight and length were measured for all 542 neonates while other anthropometric measurements were available for 266 neonates as these measurements began to be taken later in the study. Waist:hip, waist:length and subscapular skinfold:triceps skinfold ratios were calculated as were sum of triceps and subscapular skinfold thicknesses and sum of all skinfold thicknesses were in order to measure neonatal adiposity. The most commonly reported anthropometric parameters, “weight” and “length”, are very limited measures of adiposity which give no information on body fat distribution. Therefore, the more in-depth measurements above were used. Circumferences and skinfolds describe body weight distribution with skinfolds giving a measure of subcutaneous fat. Multiple-site skinfolds have been found to be more accurate than single site skinfolds and subscapular-to-triceps skinfold ratio measurement has been found to be reflective of central adiposity in children and correlates well with BMI and waist circumference [18, 19]. Waist:height ratio has been found to be a good measure of central adiposity in adults and children with a ratio of ≥0.5 indicating excess central adiposity [20]. A study by Brambilla *et al.* found it to be a better measure of adiposity than waist circumference or BMI in children and adolescents [21]. Waist circumference to height ratio has also been found to be capable of identifying children with increased cardiometabolic risk factors with some studies [22, 23] finding it a better identifier of cardiovascular disease risk than BMI.

### Maternal dietary intake

Three-day food diaries were completed at each trimester of pregnancy and used to determine maternal energy and micronutrient intake. Micronutrient intake during each trimester of pregnancy was examined separately and adjusted for maternal energy intake.

All food diaries and food frequency questionnaires were entered by a trained dietitian with the use of the household measures and UK Food Standards Agency average portion sizes [24]. Food Diaries were analysed using Tinuviel WISP software, version 3.0, in which the food composition tables used are derived from the 6th edition of McCance and Widdowson’s Food Composition Tables. Underreporting was examined using Goldberg ratios i.e. the ratio of energy intake to estimated basal metabolic rate. Basal metabolic rate was calculated using Schofield equations and a Goldberg ratio of ≤0.9 was used to identify definite underreporters [25, 26]. Vitamin and mineral supplement use was reported as a binary yes/no answer in questionnaires.

### Statistical analysis

Statistical analysis was completed using SPSS (Statistical Package for the Social Sciences) software version 20.0. Statistical analyses involved correlations, independent sample t-tests and ANOVA and simple and multiple linear regression modelling. The intervention and control groups were analysed both separately and together to ensure all results were representative of both groups. Since there was no difference in neonatal anthropometry except for thigh circumference [27] and waist:length ratio between the control and intervention groups, groups were analysed together for final analysis but group was controlled for in all final models. Micronutrients were examined per 10 MJ energy. Associations between macro- and micro-nutrients and neonatal anthropometry were first examined using correlations. Variables that were found to be significantly associated with neonatal anthropometry were further analysed using simple linear regression, then input into the final multiple regression model for well-being using a forced enter and backwards stepwise approach. While the focus of this analysis was to examine the associations between maternal micronutrient intakes and neonatal adiposity, macronutrients were also examined in order to determine whether micronutrients were independently associated with offspring adiposity or were simply acting as markers of macronutrient intakes. Both macronutrients and micronutrients that were statistically significantly associated with neonatal anthropometry using simple linear regression were included in a backwards stepwise multiple regression block resulting in any non-significant variables being discarded from the model in a stepwise manner. Variables known to affect neonatal size (maternal education level as a marker of socioeconomic status, pre-pregnancy BMI, length of gestation and neonate gender), were controlled for using a forced enter multiple regression block in all models. As mentioned, membership of the control or intervention group was also included in these models. Underreporting of dietary intake was addressed by removing definite underreporters (Goldberg ratio ≤0.9) from the multiple linear regression analysis. Supplement use was also controlled for in the final multiple linear regression models. Multiple linear regression resulted in a best and final model and models that were statistically significant overall (*p* <0.05) were chosen as those which best predicted neonatal adiposity. Compliance with Irish recommended dietary allowances [28] was also examined using the population compliance method [29].

### Sample size for maternal micronutrient effect on neonatal skinfolds

We used information from a previous publication [30] to obtain a standard deviation for a sum of skinfolds (SS+TR) measure, and to obtain regression analysis effect sizes for a generic standardised predictor variable (SD = 1). The sample sizes to detect an effect in linear regression of SS+TR on a generic micronutrient level are presented below. The predictor (micronutrient) is assumed standardised to have a standard deviation of 1. The slope therefore reflects the detectable difference in SS+TR, for a 1 SD difference in the predictor, at a power of 80 % with a type I error rate of 0.05:

**Table:** 

Slope (mm)	Sample size (total)
0.5	575
1	145
1.5	66
2	38

From Donahue *et al.*, the observed effect of a 1 SD difference in each predictor on SS+TR reached as high as 0.64 (Total n-6 fatty acids), 0.81 (n-6:n-3 ratio), and 0.61 (LA:ALA ratio) all of which should be detectable in a sample of at least 387 subjects.

## Results

### Demographics

Maternal characteristics are displayed in Table 1 with a comparison of participants and those lost to follow-up. 91.4 % of the 542 women were of “white Irish”, 6.7 % of “white other”, 0.3 % of “African”, 0.5 % of “Chinese”, 0.1 % of “Indian” and 1.0 % of “Filipino/South East Asian” ethnicity. 78.1 % of the women had achieved 3rd level education while 21.9 % had not. 4.0 % of women reported smoking in early pregnancy while 96.0 % reported being non-smokers. Participants were significantly older and had a lower early pregnancy weight and BMI than those lost to follow up but there was no difference in gestational weight gain or neonatal anthropometry between the groups. A comparison of maternal and infant characteristics of infants with and without circumference and skinfold measurements was also made (Table 2). No difference in maternal characteristics or neonatal weight or length was observed between those with and without these measurements.

**Table 1:** General maternal background characteristics during pregnancy and neonatal anthropometry with comparison of participants and those that were lost to follow-up

		Lost to follow-up			Participated		
	N	Mean	SD	n	Mean	SD	*p*
Mother age (yrs)	222	32.17	4.75	483	32.87	3.93	0.041
Mother height (cm)	240	165.10	16.58	542	165.27	11.86	0.868
Mother weight (kg)	238	76.48	16.52	541	72.38	12.94	<0.001
Mother BMI^a^ (kg/m^2^)	237	27.61	6.01	540	26.30	4.38	0.001
Gestational Weight Gain (kg)	93	12.87	5.07	273	13.39	4.55	0.359
Days per week walking ≥30 min^b^	94	3.43	1.81	427	3.48	1.79	0.772
Moderate activity (min per week)	57	59.65	43.01	309	66.15	43.45	0.299
Neonatal weight (kg)	207	4.01	604.18	542	4.02	4.58	0.633
Neonatal length (cm)	184	52.74	2.56	454	52.75	2.33	0.964
Neonatal MUAC^c^ (cm)	43	12.57	1.23	223	12.39	1.51	0.475
Neonatal abdominal circ^d^ (cm)	43	33.47	2.26	222	33.40	2.11	0.844
Neonatal waist circ^d^:hip ratio	42	33.43	2.27	221	33.63	2.32	0.606
Neonatal thigh circ^d^ (cm)	43	16.23	1.44	222	16.16	1.67	0.801
Neonatal biceps skinfold (mm)	33	6.85	1.72	186	6.78	1.46	0.806
Neonatal triceps skinfold (mm)	33	6.94	1.50	186	7.00	1.52	0.822
Neonatal subscapular skinfold (mm)	33	7.14	2.07	186	6.90	1.49	0.423
Neonatal thigh skinfold (mm)	33	8.08	2.20	186	7.90	1.76	0.605
Neonatal waist:length circ^d^ ratio	37	0.63	0.04	182	0.64	0.05	0.775
Neonatal SOSF^e^ (mm)	33	29.00	6.45	186	28.58	5.18	0.677
Neonatal sum TSF^f^ and SSF^g^ (mm)	33	14.08	3.19	186	13.90	2.69	0.736
Neonatal SSF^g^:TSF^f^ ratio	33	1.04	0.23	186	1.00	0.19	0.332
Neonatal waist:hip circ^d^ ratio	42	1.00	0.05	221	1.00	0.06	0.312

“n” denotes number, *p* <0.05 was considered statistically significant (2-tailed significance generated from independent sample t-tests)

*Abbreviations:*^a^*BMI* body mass index, ^b^*min* minutes, ^c^*MUAC* mid-upper arm circumference, ^d^*circ* circumference, ^e^*SOSF* sum of skinfolds, ^f^*TSF* triceps skinfold, ^g^*SSF* subscapular skinfold

**Table 2:** Comparison of maternal and offspring characteristics between participants with and without skinfold measurements

	No skinfolds			Skinfolds			
	n	Mean	SD	n	Mean	SD	*P*
Mother age (yrs)	309	32.89	3.82	176	32.85	4.11	0.905
Mother height (cm)	321	165.71	11.32	227	164.66	12.45	0.304
Mother weight (kg)	322	72.21	13.05	226	72.81	12.86	0.591
Mother BMI^1^ (kg/m^2^)	320	26.12	4.39	226	26.61	4.40	0.200
Gestational weight gain (kg)	152	13.18	4.37	121	13.65	4.78	0.399
Days per week walking ≥30 min^2^	255	3.51	1.85	178	3.43	1.71	0.637
Moderate activity (min per week)	191	65.97	44.67	122	65.74	41.50	0.963
Birth_weight (kg)	319	4.00	4.55	223	4.06	459.42	0.127
BirthLength (cm)	282	52.66	2.44	176	52.89	2.18	0.316

*Abbreviations:*^1^Body Mass Index, ^2^Minutes

### Maternal dietary intake and underreporting

Adequacy of dietary maternal micronutrient intake in each trimester when compared to Irish RDA’s for pregnancy [28] is displayed in Table 3 The percentage of participants who met the RDA’s for pregnancy was particularly low for folate, vitamin C and vitamin D (9.8 % in trimester 1, 21.3 % in trimester 2 and 7.2 % in trimester 3 respectively). Maternal dietary intakes and a comparison of study groups is displayed in Additional file 1: Table S1. Underreporting was found to affect the associations observed in the final multiple linear regression models. Therefore underreporters were removed from this analysis (Table 4).

**Table 3:** Micronutrient RDAs for pregnancy and proportion of women meeting these RDAs with comparison of frequency of meeting RDAs between the control and intervention groups

	RDA	n meeting RDA	% meeting RDA	*p*-value
Trimester 1 Riboflavin	1.3 mg/d	460	100	1.0
Trimester 2 Riboflavin	1.6 mg/d	466	100	1.0
Trimester 3 Riboflavin	1.6 mg/d	461	100	1.0
Trimester 1 Thiamine	100ug/MJ	460	100	1.0
Trimester 2 Thiamine	100ug/MJ	466	100	1.0
Trimester 3 Thiamine	100ug/MJ	461	100	1.0
Trimester 1 Niacin	1.6 mg/MJ	460	100	1.0
Trimester 2 Niacin	1.6 mg/MJ	466	100	1.0
Trimester 3 Niacin	1.6 mg/MJ	461	100	1.0
Trimester 1 Vitamin C	60 mg/d	238	51.7	0.008
Trimester 2 Vitamin C	80 mg/d	121	26.0	0.026
Trimester 3 Vitamin C	80 mg/d	98	21.3	0.189
Trimester 1 Vitamin B6	15ug/g protein	460	100	1.0
Trimester 2 Vitamin B6	15ug/g protein	466	100	1.0
Trimester 3 Vitamin B6	15ug/g protein	461	100	1.0
Trimester 1 Folate	500ug/d	45	9.8	0.077
Trimester 2 Folate	500 ug/d	37	7.9	0.594
Trimester 3 Folate	500 ug/d	54	11.7	0.145
Trimester 1 Vitamin B12	1.4 ug/d	460	100	1.0
Trimester 2 Vitamin B12	1.6 ug/d	466	100	1.0
Trimester 3 Vitamin B12	1.6 ug/d	461	100	1.0
Trimester 1 Vitamin D	10 ug/d	8	1.7	N/A*
Trimester 2 Vitamin D	10 ug/d	12	2.6	N/A*
Trimester 3 Vitamin D	10 ug/d	33	7.2	0.965
Trimester 1 Calcium	800 mg/d	460	100	1.0
Trimester 2 Calcium	1200 mg/d	228	48.9	0.809
Trimester 3 Calcium	1200 mg/d	264	57.3	0.482
Trimester 1 Potassium	3100 mg/d	335	72.8	0.449
Trimester 2 Potassium	3100 mg/d	368	80.0	0.010
Trimester 3 Potassium	3100 mg/d	373	80.9	0.004
Trimester 1 Iron	14 mg/d	247	53.7	0.003
Trimester 2 Iron	15 mg/d	178	38.2	0.028
Trimester 3 Iron	15 mg/d	190	41.2	0.021
Trimester 1 Zinc	7 mg/d	460	100	1.0
Trimester 2 Zinc	7 mg/d	466	100	1.0
Trimester 3 Zinc	7 mg/d	461	100	1.0
Trimester 1 Selenium	55 ug/d	299	65.0	0.005
Trimester 2 Selenium	55 ug/d	297	63.7	0.005
Trimester 3 Selenium	55 ug/d	274	59.4	0.036
Trimester 1 Iodine	130 ug/d	460	100	1.0
Trimester 2 Iodine	130 ug/d	466	100	1.0
Trimester 3 Iodine	130 ug/d	461	100	1.0
Trimester 1 Sodium	2400 mg/d	353	76.7	0.131
Trimester 2 Sodium	2400 mg/d	364	78.1	0.365
Trimester 3 Sodium	2400 mg/d	335	72.7	0.003
Trimester 1 Vitamin A	600 ug/d	119	25.9	<0.001
Trimester 2 Vitamin A	700 ug/d	48	10.3	0.881
Trimester 3 Vitamin A	700 ug/d	119	25.8	<0.001

Based on Irish RDAs [28]. Insufficient data to provide recommendations for: ß Carotene and other carotenoids Pantothenic acid, Vitamin K, Magnesium, Molybdenum, Manganese, Biotin, Fluoride, Chloride, Chromium and Vitamin E. Definite underreporters excluded (Goldberg Ratio ≤0.9). The population compliance method was used to determine frequency of compliance with RDAs [35]. Chi-squared tests used to compare frequency of compliance with RDAs between the control and intervention groups. *N/A as zero women in the intervention group met the RDA for vitamin D in trimesters 1 or 2

**Table 4:** Maternal micronutrient intakes associated with neonatal anthropometry- final models

	B	SEB	*p*	R^2^adj	f	*p*
Birthweight						
Trimester 2 vitamin D/10 MJ	−19.62	6.558	0.003	0.198	13.19	<0.001
Trimester 3 vitamin B12/10 MJ	104.73	33.349	0.002			
Birth length						
Trimester 3 magnesium/10 MJ	0.01	0.002	0.010	0.129	8.06	<0.001
Abdominal circumference						
Trimester 3 selenium/10 MJ	−0.02	0.008	0.028			
Trimester 3 retinol/10 MJ	0.001	0.001	0.020	0.119	2.93	0.002
Trimester 3 vitamin E/10 MJ	−0.11	0.053	0.039			
Thigh circumference						
Trimester 3 magnesium/10 MJ	−0.01	0.002	0.018	0.052	2.16	0.042
Hip circumference						
Trimester 3 vitamin B6/10 MJ	1.05	0.329	0.002	0.100	3.31	0.003
Mid-upper arm circumference						
Trimester 2 sodium/10 MJ	−0.0002	0.0001	0.081			
Trimester 2 vitamin B6/10 MJ	−0.23	0.052	<0.001	0.260	6.58	
Trimester 3 selenium/10 MJ	−0.01	0.005	0.002			<0.001
Subscapular skinfold thickness						
Trimester 1 vitamin C/10 MJ	0.004	0.002	0.024	0.088	2.39	0.021
Trimester 1 vitamin B12/10 MJ	0.50	0.246	0.045			
Subscapular:triceps skinfold ratio						
Trimester 1 selenium/10 MJ	−0.002	0.001	0.026			
Trimester 2 iodine/10 MJ	0.0003	0.0002	0.092	0.072	2.00	0.047
Trimester 2 retinol/10 MJ	9.632E-05	0.00005	0.050			
Waist circumference:length ratio						
Trimester 2 magnesium/10 MJ	−0.0003	0.0001	0.001	0.201	3.92	<0.001

Multiple linear regression analysis controlling for maternal education, pre-pregnancy BMI, length of gestation (d), neonate gender, study group (intervention/control), supplement use (yes/no) and significantly associated macronutrient intake (%TE). Definite underreporters (Goldberg ratio ≤0.9) removed. *p* <0.05 considered statistically significant

### Associations between maternal micronutrient intake and neonatal anthropometry

Statistically significant multiple linear regression models for the association between maternal micronutrient intakes and neonatal anthropometry are displayed in Table 4 and were as follows: birthweight was negatively associated with maternal trimester 3 vitamin D intake and positively associated with trimester 3 vitamin B12 intake R2adj 19.8 % (F = 13.19, *p* <0.001), birth length was positively associated with trimester 3 magnesium intake R2adj 12.9 % (F = 8.06, *p* <0.001), neonatal abdominal circumference was positively associated with maternal trimester 3 retinol intake and negatively associated with trimester 3 vitamin E and selenium intake R2adj 11.9 % (F = 2.93, *p* = 0.002), neonatal waist:length ratio was negatively associated with trimester 3 magnesium intake R2adj 20.1 % (F = 3.92, *p* <0.001), SSF:TSF ratio was negatively associated with trimester 1 selenium intake R2adj7.2 % (F = 2.00, *p* = 0.047). There was no association between reported maternal vitamin or mineral supplement use during pregnancy and any of the neonatal anthropometric measurements, ratios or sums of skinfold thicknesses and therefore all micronutrient associations described are from dietary intake alone.

## Discussion

The main findings of this analysis which examined the associations between neonatal anthropometry and micronutrient intakes were that birthweight was negatively associated with trimester 3 vitamin D intake and positively associated with trimester 3 vitamin B12 intake while birth length was positively associated with trimester 3 vitamin B12 intake. Abdominal circumference was positively associated with maternal trimester 3 retinol intake and negatively associated with trimester 3 vitamin E and selenium intake. When ratios for estimation of neonatal adiposity were calculated, it was found that the subscapular:triceps skinfold thickness ratio, a measure of central adiposity, was negatively associated with trimester 1 selenium intake while the waist circumference:length ratio, another measure of central adiposity, was negatively associated with trimester 3 magnesium intake. Our finding that birthweight was negatively associated with trimester 3 vitamin D intake is contrary to to the findings other studies [31, 32]. This may be due, in part, to the nature of our cohort, in which there was a high prevalence of macrosomia as a US randomised control trial of vitamin D supplementation in pregnancy by Wagner *et al.* [33] found that, when macrosomia was examined, the group supplemented with the highest doses of vitamin D gave birth to significantly more infants of normal birthweight than those supplemented with lower doses.

Maternal trimester 3 vitamin B12 intake was positively associated with birthweight. This effect on fetal growth is thought to be due to the activity of vitamin B12 as a coenzyme in the conversion of homocysteine to methionine which is in turn converted to S-adenosylmethionine. In vitamin B12 deficiency, this reaction is reduced resulting in increased levels of homocysteine which alters the placental vascular endothelium leading to fetal growth restriction [34, 35]. Levels of S-adenosylmethionine are also concomitantly reduced, decreasing the level of DNA methylation and are thereby thought to epigenetically reduce fetal growth [36, 37]. Study results been conflicting regarding the association of vitamin B12 and birthweight with some reporting a positive association [38–41] and others observing no such association [42–46]. A recent study which did not observe an association between birth weight or length and maternal vitamin B12 levels found that when the ratio of maternal plasma folate to vitamin B12 was examined there was an inverse association with neonatal weight, length, head circumference and chest circumference but no association was observed with neonatal triceps or subscapular skinfold thickness [43]. No such inverse association was observed in our analysis, however. Another study reported a positive association between birthweight and absolute vitamin B12 and also observed a further adverse effect on birthweight of folate supplementation in women with poor vitamin B12 status [38]. Therefore, increased folate levels resulting in increased homocysteine levels when vitamin B12 is rate-limiting may negatively affect birthweight.

The positive association of maternal trimester 3 magnesium intake with birth length may be due to the role of magnesium in skeletal growth with the greatest rate of mineral accretion occurring in the 3rd trimester [47]. A study by Doyle *et al.* [48] found that magnesium intake of 513 women in trimester 1 was similarly associated with birth length but that magnesium supplementation in later trimesters did not affect infant size. Similarly, the ALSPAC study found that bone mineral density and height of 9 year old children was associated with maternal magnesium intake during late pregnancy [49] and another Tasmanian study found the bone mass of 8 year old children was positively associated with maternal magnesium intake in trimester 3 [50]. This was hypothesised however to be mediated by fetal programming of growth by maternal diet during pregnancy rather than by fetal bone mineralisation [49, 50]. The finding that trimester 2 magnesium intake was negatively associated with neonatal waist:length circumference, a marker of central adiposity [20], may also due to an association with increased neonatal length. A further possible explanation for this association may be that is a maker of overall healthy diet [51] although maternal BMI and macronutrient intake was controlled for in all of our final models. In addition, there is also evidence that low blood magnesium levels and magnesium deficiency are associated with inflammation in adults and children [52–54], and with insulin insensitivity and metabolic syndrome and consequently central adiposity [52, 55].

Selenium was negatively associated with two measures of central adiposity, trimester 3 selenium with the abdominal circumference and trimester 1 selenium with the subscapular:triceps skinfold ratio. The main function of selenium is as an antioxidant due to its incorporation into selenoproteins which have are involved in glutathione peroxidase production and many other anti-inflammatory mechanisms, not all of which are yet understood [56]. Increased selenium levels may modulate selenoprotein gene expression and selenium supplementation has been found to be associated with reduced TNF-α levels *in vitro* and *in vivo* [57, 58]. However, selenium can also act through modulating the effect of TNF-α via TNF-α receptors [59] and can up-regulate production of TNF-α in individuals in whom it is under-produced for example individuals at risk of carcinoma [60]. It therefore acts as an anti-proliferative agent and is also a component of many enzymes in the body. Low selenium status in pregnancy has been linked with reduced birthweight [61–63] and with gestational diabetes and maternal glucose intolerance [64–66]. Although we found that dietary selenium was negatively associated with central adiposity in this cohort, we did not observe an association with birthweight. Studies exploring the relationship between selenium and birthweight, unlike this study, have examined serum rather than dietary selenium levels and have proposed that oxidative stress in these pregnancies may have resulted in selenium depletion.

Finally, maternal trimester 2 and 3 retinol intakes were positively associated with central adiposity. Low maternal retinol status has been found to be associated with reduced offspring birthweight by several studies [67–70] although other studies have reported opposing results [71–74]. However, as with all micronutrients associated with neonate adiposity in this cohort, the association with neonatal adiposity specifically, rather than just birthweight, has not been reported in the literature to the best of the authors’ knowledge.

Limitations of this study were that blood micronutrient levels were not measured although extensive dietary records were obtained from 3-day food diaries at each trimester of pregnancy. Detailed information on micronutrient supplementation in pregnancy was not available as this was a secondary analysis. A further limitation was that we were unable to rule out the possibility of micronutrients synergistic activity as mentioned above. Equally, it is possible that micronutrients may have been acting as markers of improved maternal health behaviours [10] although we attempted to reduce the likelihood of this by controlling for BMI and controlling for any macronutrients associated with neonatal anthropometry in all final models. Finally, an additional limitation was that circumference and skinfold thickness measurements were not available for the full cohort resulting in reduced statistical power of the analysis. No significant difference in maternal or offspring characteristics was observed when infants with and without circumference and skinfold measurements were compared, however. There has been little research into intakes of micronutrients in overweight and obese pregnant women with no studies specifically exploring the association with offspring adiposity and the ROLO study was appropriate for exploration of these associations as there was a high level of overweight (mean BMI in early pregnancy 26.3 kg/m2) and macrosomia (mean birthweight of 4.02 kg) in this cohort. Further research is necessary to define such relationships and determine optimum maternal micronutrient intakes for overweight or obese pregnancies.

## Conclusions

Dietary intakes of several micronutrients were poor during pregnancy in women from a developed country. Maternal dietary micronutrient intake is associated with neonatal anthropometry even in women not at risk of malnutrition and further research is necessary to determine optimal micronutrient intake in overweight and obese women.

## Additional file

Additional file 1: Table S1. Maternal Energy and Micronutrient Intakes in Each Trimester of Pregnancy including Comparison of Control and Low Glycaemic Index Intervention Groups. (DOC 119 kb)
