# Supplementary material for: The association between maternal dietary micronutrient intake and neonatal anthropometry – secondary analysis from the ROLO study
Source: Nutr J. 2015 Oct 7;14:105. doi: 10.1186/s12937-015-0095-z (PMC4597429; doi:10.1186/s12937-015-0095-z)
Supplement: Additional file 1: Table S1. — Maternal Energy and Micronutrient Intakes in Each Trimester of Pregnancy including Comparison of Control and Low Glycaemic Index Intervention Groups. (DOC 119 kb) [file 12937_2015_95_MOESM1_ESM.doc]

Supplementary Table 1. Maternal Energy and Micronutrient Intakes in Each Trimester of Pregnancy including Comparison of Control and Low Glycaemic Index Intervention Groups.

|  | **N** | **Intervention** | **Control** | **Total** | **p-value** |
| --- | --- | --- | --- | --- | --- |
| T1 Energy Intake (kcal/d) | 541 | 1828.25±407.98 | 1874.02±474.10 | 1854.47±446.12 | 0.245 |
| T2 Energy Intake (kcal/d) | 547 | 1803.43±440.82 | 1943.24±476.75 | 1883.50±467.54 | 0.001 |
| T3 Energy Intake (kcal/d) | 542 | 1832.55±424.02 | 1932.42±472.56 | 1889.62±454.91 | 0.011 |
| T1 Sodium (mg/d) | 541 | 2587.27±761.86 | 2719.16±812.16 | 2660.46±791.38 | 0.054 |
| T2 Sodium (mg/d) | 547 | 2583.79±816.57 | 2693.17±826.15 | 2650.72±835.04 | 0.123 |
| T3 Sodium (mg/d) | 542 | 2625.42±714.65 | 2717.25±834.31 | 2675.82±783.56 | 0.176 |
| T1 Potassium (mg/d) | 541 | 2755.63±773.92 | 2676.83±748.40 | 2714.76±762.21 | 0.231 |
| T2 Potassium (mg/d) | 547 | 2744.98±740.46 | 2775.68±715.13 | 2766.57±733.12 | 0.624 |
| T3 Potassium (mg/d) | 542 | 2769.87±727.72 | 2764.89±730.95 | 2772.00±737.06 | 0.937 |
| T1 Calcium (mg/d) | 541 | 912.36±315.88 | 890.02±312.66 | 899.22±314.22 | 0.412 |
| T2 Calcium (mg/d) | 547 | 915.17±334.23 | 907.37±299.88 | 911.03±315.10 | 0.774 |
| T3 Calcium (mg/d) | 542 | 960.15±355.20 | 926.82±321.87 | 941.09±337.06 | 0.254 |
| T1 Magnesium (mg/d) | 541 | 253.45±67.65 | 242.78±71.22 | 247.77±69.93 | 0.077 |
| T2 Magnesium (mg/d) | 547 | 259.43±72.91 | 249.07±67.74 | 254.38±72.04 | 0.087 |
| T3 Magnesium (mg/d) | 542 | 262.84±72.12 | 247.40±66.61 | 255.07±71.97 | 0.010 |
| T1 Iron (mg/d) | 541 | 11.03±3.38 | 11.22±3.79 | 11.14±3.61 | 0.528 |
| T2 Iron (mg/d) | 547 | 11.14±3.38 | 11.27±3.45 | 11.24±3.47 | 0.651 |
| T3 Iron (mg/d) | 542 | 11.21±3.44 | 11.34±3.60 | 11.30±3.55 | 0.675 |
| T1 Iodine (ug/d) | 541 | 129.38±70.53 | 128.48±61.75 | 128.75±65.76 | 0.875 |
| T2 Iodine (ug/d) | 547 | 133.59±60.38 | 136.64±66.43 | 135.14±63.80 | 0.580 |
| T3 Iodine (ug/d) | 542 | 152.07±85.15 | 139.60±69.37 | 145.01±76.94 | 0.061 |
| T1 Vitamin D (ug/d) | 541 | 2.53±1.65 | 2.57±2.01 | 2.54±1.86 | 0.802 |
| T2 Vitamin D (ug/d) | 547 | 2.87±1.97 | 2.70±2.13 | 2.78±2.06 | 0.337 |
| T3 Vitamin D (ug/d) | 542 | 3.07±2.85 | 2.81±2.76 | 2.92±2.80 | 0.278 |
| T1 Vitamin K (ug/d) | 541 | 118.69±83.27 | 117.75±81.54 | 118.47±82.46 | 0.894 |
| T2 Vitamin K (ug/d) | 547 | 112.23±75.41 | 111.73±74.11 | 112.18±74.73 | 0.938 |
| T3 Vitamin K (ug/d) | 542 | 107.99±74.11 | 116.78±81.69 | 113.17±78.69 | 0.196 |
| T1 Vitamin B6 (mg/d) | 541 | 1.53±0.45 | 1.55±0.50 | 1.54±0.48 | 0.654 |
| T2 Vitamin B6 (mg/d) | 547 | 1.61±1.52 | 1.58±0.44 | 1.66±1.92 | 0.721 |
| T3 Vitamin B6 (mg/d) | 542 | 1.61±1.14 | 1.59±0.46 | 1.62±1.00 | 0.756 |
| T1 Vitamin B12 (ug/d) | 541 | 1.62±0.55 | 1.63±0.56 | 1.63±0.56 | 0.887 |
| T2 Vitamin B12 (ug/d) | 547 | 1.67±0.54 | 1.70±0.56 | 1.69±0.55 | 0.513 |
| T3 Vitamin B12 (ug/d) | 542 | 1.78±0.65 | 1.75±0.58 | 1.77±0.61 | 0.555 |
| T1 Thiamine (mg/d) | 541 | 2.08±0.68 | 2.09±0.72 | 2.08±0.70 | 0.888 |
| T2 Thiamine (mg/d) | 547 | 2.10±0.67 | 2.16±0.67 | 2.13±0.67 | 0.271 |
| T3 Thiamine (mg/d) | 542 | 2.08±0.66 | 2.15±0.71 | 2.12±0.69 | 0.216 |
| T1 Riboflavin (mg/d) | 541 | 2.00±0.56 | 2.02±0.67 | 2.01±0.62 | 0.630 |
| T2 Riboflavin (mg/d) | 547 | 1.99±0.55 | 2.11±0.62 | 2.06±0.59 | 0.016 |
| T3 Riboflavin (mg/d) | 542 | 1.99±0.57 | 2.14±0.64 | 2.07±0.62 | 0.007 |
| T1 Niacin (mg/d) | 541 | 40.70±19.50 | 42.70±20.11 | 41.80±20.40 | 0.255 |
| T2 Niacin (mg/d) | 547 | 44.00±20.30 | 43.10±20.60 | 43.50±20.40 | 0.636 |
| T3 Niacin (mg/d) | 542 | 47.20±25.70 | 47.10±30.00 | 47.10±28.20 | 0.993 |
| T1 Folate (ug/d) | 541 | 277.04±98.35 | 266.94±107.49 | 271.86±103.88 | 0.260 |
| T2 Folate (ug/d) | 547 | 273.49±92.44 | 270.44±94.24 | 272.07±93.51 | 0.704 |
| T3 Folate (ug/d) | 542 | 277.04±102.63 | 274.43±106.83 | 275.77±104.89 | 0.774 |
| T1 Pantothenic Acid (mg/d) | 541 | 4.94±1.60 | 4.86±1.44 | 4.90±1.51 | 0.552 |
| T2 Pantothenic Acid (mg/d) | 547 | 4.96±1.51 | 5.09±1.50 | 5.04±1.50 | 0.322 |
| T3 Pantothenic Acid (mg/d) | 542 | 5.18±1.57 | 5.16±1.48 | 5.17±1.52 | 0.892 |
| T1 Biotin (ug/d) | 541 | 27.81±9.42 | 27.45±10.21 | 27.60±9.85 | 0.673 |
| T2 Biotin (ug/d) | 547 | 29.01±11.10 | 28.34±10.91 | 2 8.69±11.04 | 0.481 |
| T3 Biotin (ug/d) | 542 | 30.56±11.93 | 28.50±10.23 | 29.42±11.05 | 0.032 |
| T1 Zinc (mg/d) | 541 | 8.36±2.45 | 8.42±2.60 | 8.40±2.53 | 0.799 |
| T2 Zinc (mg/d) | 547 | 8.78±2.93 | 8.71±2.56 | 8.77±2.81 | 0.767 |
| T3 Zinc (mg//d) | 542 | 8.86±2.39 | 8.52±2.64 | 8.68±2.55 | 0.125 |
| T1 Selenium (ug/d) | 541 | 44.74±18.40 | 44.65±19.52 | 44.66±19.01 | 0.959 |
| T2 Selenium (ug/d) | 547 | 44.09±16.00 | 45.15±17.44 | 44.72±16.82 | 0.474 |
| T3 Selenium (ug/d) | 542 | 44.28±16.22 | 43.61±17.15 | 43.92±16.72 | 0.645 |
| T1 Retinol (ug/d) | 541 | 319.81±149.66 | 339.74±330.18 | 330.38±266.07 | 0.396 |
| T2 Retinol (ug/d) | 547 | 310.91±181.98 | 326.70±184.25 | 319.27±183.41 | 0.325 |
| T3 Retinol (ug/d) | 542 | 338.28±189.88 | 375.24±594.80 | 358.38±461.04 | 0.355 |
| T1 Carotene (ug/d) | 541 | 3526.81±2462.03 | 3241.61±2381.07 | 3365.24±2417.20 | 0.182 |
| T2 Carotene (ug/d) | 547 | 3422.47±2498.64 | 3229.78±2695.06 | 3315.75±2607.35 | 0.400 |
| T3 Carotene (ug/d) | 542 | 3531.43±2799.34 | 3177.52±2660.97 | 3353.13±2758.07 | 0.134 |
| T1 Vitamin E (ug/d) | 541 | 8.33±3.91 | 7.93±3.62 | 8.11±3.75 | 0.223 |
| T2 Vitamin E (ug/d) | 547 | 8.33±3.70 | 8.15±3.62 | 8.24±3.66 | 0.566 |
| T3 Vitamin E (ug/d) | 542 | 8.18±3.41 | 8.23±3.64 | 8.23±3.58 | 0.878 |
| T1 Vitamin C (mg/d) | 541 | 30.05±36.17 | 34.47±63.32 | 32.64±53.09 | 0.346 |
| T2 Vitamin C (mg/d) | 547 | 27.87±32.38 | 30.14±43.88 | 29.12±39.18 | 0.509 |
| T3 Vitamin C (mg/d) | 542 | 26.41±33.77 | 27.63±39.51 | 27.09±37.01 | 0.703 |

T, trimester, p<0.05 was considered statistically significant. 2-tailed significance generated from independent sample t-tests
